# A First Study of the Virulence Potential of a *Bacillus subtilis* Isolate From Deep-Sea Hydrothermal Vent

**DOI:** 10.3389/fcimb.2019.00183

**Published:** 2019-05-31

**Authors:** Han-Jie Gu, Qing-Lei Sun, Jing-Chang Luo, Jian Zhang, Li Sun

**Affiliations:** ^1^CAS Key Laboratory of Experimental Marine Biology, Center for Ocean Mega-Science, Institute of Oceanology, Chinese Academy of Sciences, Qingdao, China; ^2^Laboratory for Marine Biology and Biotechnology, Pilot National Laboratory for Marine Science and Technology, Qingdao, China; ^3^College of Earth and Planetary Sciences, University of Chinese Academy of Sciences, Beijing, China

**Keywords:** *Bacillus subtilis*, deep-sea, hydrothermal vent, virulence, genome

## Abstract

*Bacillus subtilis* is the best studied Gram-positive bacterium, primarily as a model of cell differentiation and industrial exploitation. To date, little is known about the virulence of *B. subtilis*. In this study, we examined the virulence potential of a *B. subtilis* strain (G7) isolated from the Iheya North hydrothermal field of Okinawa Trough. G7 is aerobic, motile, endospore-forming, and requires NaCl for growth. The genome of G7 is composed of one circular chromosome of 4,216,133 base pairs with an average GC content of 43.72%. G7 contains 4,416 coding genes, 27.5% of which could not be annotated, and the remaining 72.5% were annotated with known or predicted functions in 25 different COG categories. Ten sets of 23S, 5S, and 16S ribosomal RNA operons, 86 tRNA and 14 sRNA genes, 50 tandem repeats, 41 mini-satellites, one microsatellite, and 42 transposons were identified in G7. Comparing to the genome of the *B. subtilis* wild type strain NCIB 3610^T^, G7 genome contains many genomic translocations, inversions, and insertions, and twice the amount of genomic Islands (GIs), with 42.5% of GI genes encoding hypothetical proteins. G7 possesses abundant putative virulence genes associated with adhesion, invasion, dissemination, anti-phagocytosis, and intracellular survival. Experimental studies showed that G7 was able to cause mortality in fish and mice following intramuscular/intraperitoneal injection, resist the killing effect of serum complement, and replicate in mouse macrophages and fish peripheral blood leukocytes. Taken together, our study indicates that G7 is a *B. subtilis* isolate with unique genetic features and can be lethal to vertebrate animals once being introduced into the animals by artificial means. These results provide the first insight into the potential harmfulness of deep-sea *B. subtilis*.

## Introduction

*Bacillus* species are aerobic, rod-shaped bacteria that stain Gram-positive or Gram-negative (Cote et al., 2015). They form spores that are resistant to cold, heat, and common disinfectants, thus enabling the bacteria to survive in various environments (Brown, 2000; Cote et al., 2015). *Bacillus* is a large genus with more than 200 species (Euzéby, 1997). The majority of *Bacillus* are non-pathogenic, and many species have been used for biotechnological and industrial applications (Hou et al., 2005; Price et al., 2007). Only a few species of *Bacillus* are known to cause disease in animals and humans (Spencer, 2003). Two *Bacillus* species, i.e., *Bacillus anthracis* and *Bacillus cereus*, are considered medically significant; *B. anthracis* is the etiologic agent of anthrax, a common disease of livestock, while *B. cereus* can cause food poisoning as well as local and systemic infections (Spencer, 2003; Schoeni and Wong, 2005; Hoffmaster et al., 2006; Ramarao and Sanchis, 2013). In addition, *Bacillus licheniformis* has been reported to be associated with foodborne illness (Logan, 2012), and *Bacillus thuringiensis* is an important insect pathogen (Nielsen-LeRoux et al., 2012).

Members of the genus *Bacillus* are found in diverse environments on earth including deep sea (D'Hondt et al., 2004; Batzke et al., 2007). In the deep sea hydrothermal areas, a wide range of microbes colonize, which can be free living and utilize the inorganic carbon and sulfides in the hydrothermal fields, or form various communities with host animals (Galéron, 2014). Several studies have indicated the existence of *Bacillus* species in deep sea (Marteinsson et al., 1996; Liu et al., 2006; Kurata et al., 2015; Wen et al., 2015). A report showed that, of the many isolates obtained from four different deep sea sediments, the vast majority (90%) were spore-forming bacteria related to *Bacillus* (Sass et al., 2008). Another report showed that Gram-positive, spore-forming piezophilic bacteria probably constituted a large part of cultivable deep-sea floor bacterial communities at Site C0020 off the Shimokita Peninsula, Japan, and the most abundant bacteria were members of *Bacillales* (Fang et al., 2017). In addition to being ubiquitous in deep-sea water and sediments, *Bacillus* species are shown to be present in and on marine organisms, such as sponges, ascidian, and crabs (Ivanova et al., 1999). However, to our knowledge, experimental studies on the virulence potential of *Bacillus* from deep sea have not been documented.

In this work, we reported the characterization of a *B. subtilis* strain, G7, isolated from the deep-sea hydrothermal field in Iheya North of Okinawa Trough. We analyzed the morphological, phylogenetic, genomic, and potential virulence of strain G7, and provided the first insight into the detrimental effect of *Bacillus* species from deep-sea hydrothermal vent.

## Materials and Methods

### Ethics Statement

Experiments involving live animals conducted in this study were approved by the Ethics Committee of Institute of Oceanology, Chinese Academy of Sciences. All methods were carried out in accordance with the relevant guidelines, including any relevant details.

### Experimental Animals

Clinically healthy turbot (*Scophthalmus maximus*) and half-smooth tongue sole (*Cynoglossu semilaevis*) were purchased from a commercial fish farm in Shandong Province, China and maintained at 20°C in aerated seawater. Fish were acclimatized in the laboratory for 2 weeks before experimental manipulation. Before experiment, fish were confirmed to be clinically healthy as reported previously (Hu et al., 2014) by confirmation of no bacterial presence in liver, kidney, and spleen. For tissue collection, fish were euthanized with an overdose of MS222 (tricaine methanesulfonate) (Sigma, St. Louis, USA) as reported previously (Wang et al., 2009). BALB/c mice (female, 8–10 weeks, and 14 ± 2 g) were obtained from Qingdao Daren Fortune Animal Technology Co., Ltd. Before experiment, mice were acclimatized in laboratory for 7 d under good laboratory condition (temperature 25 ± 2°C, relative humidity 50 ± 20%, unlimited access to standard pellet food and tap water, and a dark and light cycle of 12/12) as reported previously (Khasawneh et al., 2015). For tissue collection, mice were anesthetized with ketamine (80 mg/kg) (Ketavet, Pfizer, Berlin, Germany) (Dietert et al., 2017).

### Bacterial Strains and Culture Conditions

*Bacillus subtilis* subsp. *subtilis* NCIB 3610^T^ (CGMCC accession No. 1.3358) and *Bacillus subtilis* subsp. *subtilis* 168 (CGMCC accession No. 1.1390) were purchased from China General Microbiological Culture Collection Center (CGMCC, http://www.cgmcc.net).

### Isolation of G7

Strain G7 was isolated from seawater sample collected at the Iheya North hydrothermal field (126°53.84′ E, 27°47.44′ N, depth of 966.9 m, temperature of 4~5°C) in Okinawa Trough, northwestern Pacific Ocean. The seawater samples were obtained by a sample bottle (Sea-Bird O.T.E. Model 110, USA) on the Remotely Operated Vehicle (ROV) equipped on the KEXUE vessel (Tollefson, 2014). *In situ* temperature was measured using a conductivity-temperature-depth sampler. Seawater samples collected *in situ* were brought on board under totally enclosed condition and the outer surface of the sampling bottle was immediately disinfected with 75% alcohol before taking the water from the bottle; after being taken from the sampling bottle, the seawater was immediately used for bacterial isolation in an ultra-clean workbench on board as follows: 100 μl of the seawater was plated on marine agar 2216E medium (Sun et al., 2015) in an aseptic environment and incubated under aerobic conditions at 4°, 15°, 28°, or 40°C for 7 d. The colonies on the plates were screened by their shape, size, margin, color, and opacity (Valiente Moro et al., 2013). Each type of colonies was selected for purification. Ninety-six bacterial isolates were obtained, and one of which was named G7. The purified isolates were resuspended in marine 2216E medium containing 30% (v/v) glycerol and stored at −80°C.

### Phenotypic Analysis of G7

In order to determine the temperature range of growth for G7, G7 was cultured in marine 2216E medium at 4°, 20°, 37°, 50°, or 60°C for 72 h. To determine the optimal growth temperature, the growth of G7 as well as *B. subtilis* subsp*. subtilis* NCIB 3610^T^ was determined at 16°, 28°, 37°, and 50°C. To determine NaCl dependence, G7 was cultured at 28°C in marine 2216E medium containing different concentrations of NaCl (0–10%, at intervals of 0.5%). The pH range was determined from pH 4.0-11.0 (at intervals of 1.0 pH unit) using the buffer system described previously (Xu et al., 2005) in marine 2216E medium. Gram staining and spore morphology were analyzed using a Gram-staining kit and a spore staining kit (Haibo, Qingdao, China), respectively. Oxidase activity was determined using Oxidase reagent (Haibo, Qingdao, China); catalase activity was determined by bubble formation in a 10% (v/v) H_2_O_2_ solution.

### Motility Assay and Flagella Observation

Motility assay was performed as reported previously (Mi et al., 2015; Sun et al., 2016). Briefly, G7 was cultured in marine 2216E medium to an OD_600_ of 0.8, and 5 μl of cell suspension was spotted onto the center of 2216E medium plates containing 0.3% or 0.5% (w/v) agar. The plates were incubated at 28°C for 24 h, and the motility of the bacteria was observed. For flagella observation, G7 was grown in solid marine agar 2216E medium for 24 h, followed by fixation with glutaraldehyde and dehydration with acetone. The cells were then observed with a transmission electron microscopy (TEM) (Hitachi, JEM-2100, Japan) as reported previously (Sun and Sun, 2016). The assays were performed three times.

### Phylogenetic Analysis

The phylogenetic tree was constructed based on the core genes of 18 *Bacillus* sp. strains with available genome sequences (Table S1); the core genes of the 18 genomes were obtained using cd-hit 4.6.1 (Huang et al., 2010), and the corresponding protein sequences were aligned with MUSCLE 3.8.31 (Edgar, 2004); phylogenetic trees were generated with Treebest 1.9.2 (Caputo et al., 2015). The average nucleotide identity (ANI) values were calculated with EzBioCloud ANI calculator (https://www.ezbiocloud.net/tools/ani).

### Genome Sequencing and Analysis

The genomic DNA of G7 was extracted using a Bacteria DNA extraction kit (TIANGEN Biotech, Beijing, China). Genome sequencing was conducted by Novogene (Beijing, China). The sequencing was performed using the third-generation PacBio RSII platform (Pacific Biosciences, Menlo Park, USA) with 10 Kb SMRT Bell libraries, and 1.11 Gb clean data were acquired after filtering out the low-quality reads. The reads were assembled using SMRT portal assembly software (Berlin et al., 2015). Putative coding sequences were identified using GeneMarkS software (http://topaz.gatech.edu/GeneMark) (Besemer et al., 2001). Genes encoding virulence factors were identified with Virulence Factors of Pathogenic Bacteria Database (VFDB) (Chen et al., 2005). Repeated sequences were predicted with RepeatMasker (Saha et al., 2008) and TRF (Benson, 1999). rRNAs, sRNAs, and tRNAs were predicted by using RNAmmer (Lagesen et al., 2007), Rfam (Gardner et al., 2009), and tRNAscan-SE (Lowe and Eddy, 1997), respectively. The functional annotation was carried out using the BLASTP search tool (Altschul et al., 1990) and KEGG (Kyoto encyclopedia of genes and genomes; http://www.genome.jp/kegg/) (Kanehisa et al., 2004), COG (http://www.ncbi.nlm.nih.gov/COG/) (Tatusov et al., 2003), SwissProt (http://www.uniprot.org/) (Bairoch and Apweiler, 2000), GO (Gene Ontology; http://www.geneontology.org/) (Ashburner et al., 2000), and GenBank's non-redundant protein (nr) (NCBI non-redundant database; http://www.ncbi.nlm.nih.gov/RefSeq/) (Li et al., 2002) databases (parameters: minimal alignment length percentage ≥40%, identity ≥40%, e-value ≤ 1e-5). Gene Islands (GIs) were predicted with IslandPath-DIOMB (parameters: e-value = 0.001, mobility gene ≥1) (Hsiao et al., 2003). Genome mapping was generated with Circos (Krzywinski et al., 2009).

The genome sequences of G7 and *B. subtilis* subsp*. subtilis* NCIB 3610^T^ were compared using MUMmer (Kurtz et al., 2004). Predicted proteins of G7 were compared with those of NCIB 3610^T^, using BLASTP with an E-value cutoff of 1e-5. Orthologous proteins are defined as reciprocal best hit proteins with a minimum 40% identity and 70% of the length of the query protein, calculated with BLAST algorithm. Proteins without orthologs were considered to be specific proteins. COG function category was analyzed by searching all predicted proteins against the COG database on the basis of the BLASTP.

#### *In vivo* Infection and Virulence Assay

To determine tissue dissemination, G7 and strain 168 were cultured to an OD_600_ of 0.8 at 28°C in marine 2216E and LB medium, respectively; the cells were washed with PBS and resuspended in PBS to 1 × 10^6^ CFU/ml. Turbot and mice were inoculated via intramuscular (i.m., for fish) and intraperitoneal (i.p., for mice) injection with 100 μl G7 or strain 168 suspension. At 12, 24, and 48 h post-infection (hpi), blood, liver, and spleen were taken aseptically from fish (5 fish/time point) and mice (3 animals/time point). The tissues were homogenized in a homogenizer (Jingxin, Shanghai, China) containing PBST (PBS with 1% Triton X-100) (100 μl/mg tissue). The homogenates were diluted serially and plated in triplicate on marine agar 2216E plates or LB plates. The plates were incubated at 28°C for 24 h, and the colonies that appeared on the plates were enumerated. To determine median lethal dose (LD_50_), G7 was cultured in marine 2216E, NCIB 3610^T^ and strain 168 were cultured in LB, and resuspended in PBS. Fish and mice were divided randomly into groups of 20 fish or 10 mice; each group was infected via i.m. (for fish) or i.p. (for mice) injection with 100 μl bacterial suspension containing 10^4^ to 10^9^ CFU (at 10-fold difference) of bacterial cells. The animals were monitored for mortality for 14 days, and LD_50_ was determined with Probit analysis tool of the SPSS 17.0 software (SPSS Inc., USA). All experiments were conducted in three replicates.

### Serum Survival and Hemolytic Activity Assay

Serum survival analysis was performed as reported previously (Wang et al., 2013). To examine hemolytic activity tests were checked on 2% rabbit blood (Hope Bio, Qingdao, China) agar plates. G7, NCIB 3610^T^, and strain 168 were cultured in marine 2216E medium to an OD_600_ of 0.8, and 10 μl cell suspension was added onto a filter disc on a 2% rabbit blood (Hope Bio, Qingdao, China) agar plate. As controls, 0.2% Triton X-100 and PBS were also spotted similarly on the plate. The plate was incubated at 28°C for 24 h and observed for hemolytic halos.

### Intracellular Infection

Intracellular infection was performed as reported previously (Sui et al., 2017). Briefly, for infection of RAW264.7 cells, G7, NCIB 3610^T^, and strain 168 were cultured and resuspended in PBS as above; the bacteria were added to 100% confluent RAW264.7 cells (American Tissue Culture Collection, USA) in 24-well plates at a multiplicity of infection (MOI) of 10:1; the plates were centrifuged at 800 *g* for 10 min and incubated at 28°C for 2 h. The cells were then washed three times with PBS and incubated with fresh medium containing 200 μg/ml gentamicin (Thermo Scientific HyClone, Beijing, China) for 1 h to kill the extracellular bacteria. The medium was then removed, and fresh medium containing 10 μg/ml gentamicin was added to the cells. For the remaining experiment, the concentration of gentamicin in the culture medium was maintained at 10 μg/ml, and the plates were incubated at 28°C for 0, 2, 4, 6, and 8 h. For cell number counting, at each time point of incubation, fresh medium was added to the plates (500 μl/well), and the cells on the plates were scraped with a cell scraper (Costar, Corning, NY) for cell counting. For the determination of intracellular bacterial number, 500 μl 1% (v/v) Triton X-100 was added to the cells in each well of the plate to lyse the cells, and the lysate was diluted and plated onto marine 2216E agar plates, which were incubated at 28°C for 24 h, and colony-forming units (CFUs) were counted. For infection of turbot peripheral blood leukocytes (PBLs), PBLs were prepared and cultured in L-15 medium (Thermo Scientific HyClone, Beijing, China) as reported previously (Liu et al., 2010) in 96-well culture plates (10^5^ cells/ well). Intracellular infection of G7 in PBLs was performed as above. All experiments were performed three times.

### Microscopy

To prepare fluorescent bacteria, 500 μl of G7 suspension (1 × 10^8^ CFU/ml) was incubated with 5 μM CFDA-SE (US Everbright Inc, Suzhou, China) at 37°C for 20 min, followed by incubation with 5 % BSA for 20 min. The bacteria were then washed three times with PBS. For infection, Raw264.7 cells were cultured to 100% confluence in 35 mm confocal dishes (Nest, China); turbot PBLs were added to 35 mm confocal dishes (Nest, China) to 5 × 10^6^ cells/ml. The cells were then infected with G7 for 2, 4, and 6 h as above. At each time point, the cells were extensively washed with PBS and stained with Hoechst 33258 (Beyotime, Shanghai, China) for 30 min at room temperature. The cells were washed as above, and extracellular fluorescence was quenched by adding 1 ml 0.125% trypan blue in PBS, followed by incubation at 28°C for 30 min. The cells were washed as above and observed with a confocal microscope (Carl Zeiss LSM710, Germany).

### Statistical Analysis

All experiments were performed at least three times, and statistical analyses were carried out with SPSS 17.0 software (SPSS Inc., Chicago, USA). Data were analyzed with analysis of variance (ANOVA), and statistical significance was defined as *P* < 0.05.

### Database Accession Number

The whole genome sequence of G7 has been deposited in GenBank under the accession number CP029609.

## Results

### Identification and Characterization of *Bacillus subtilis* subsp. *subtilis* G7

**Figure 1:**
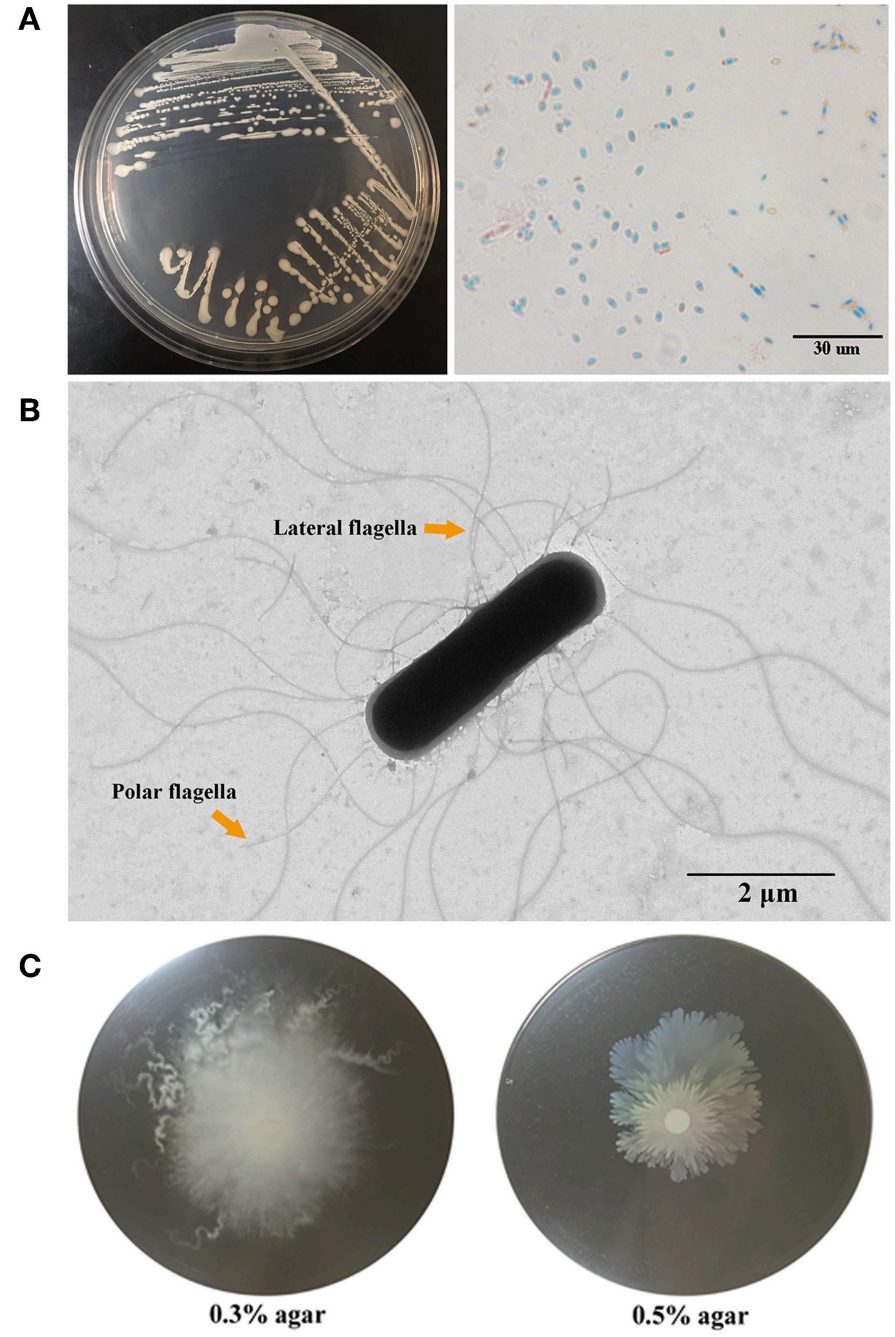
Morphology and motility of G7. **(A)** G7 colonies on Marine 2216E plate (left) and micrograph of malachite green and safranine stained spores of G7 (right). **(B)** G7 was observed with a transmission electron microscope, the arrows indicate polar and lateral flagella. **(C)** G7 suspension was spotted onto the center of marine 2216E plates containing 0.3 or 0.5% (w/v) agar, and the plates were incubated at 28°C for 24 h.

Strain G7 was isolated from the deep-sea water collected from Iheya North hydrothermal field. G7 was aerobic, motile, gram-positive, and oxidase- and catalase-positive; it required NaCl for growth and could grow at NaCl concentrations up to 8% (Figure S1). Visible growth occurred at temperatures between 20° and 60°C within a period of 3 days and in the pH range of 5–11. For G7, the optimal growths were observed at 50°, 37°, and 28°C, at which temperatures the cells grew much faster than at 16°C; the growths at 50° and 37°C were very similar and slightly faster than growth at 28°C (Figure S2). Similar to G7, NCIB 3610^T^ grew optimally at 50°, 37°, and 28°C, with the best growth occurring at 37°C, at which temperature the bacteria grew slightly faster than at 50° and 28°C (Figure S2). Colonies on marine 2216E agar medium after 24 h growth at 28°C were smooth, round, white in color, and about 3 ± 0.3 mm in diameter (Figure 1A, panel left). The cells produced endospores, which were oval and located centrally in unswollen sporangia (Figure 1A, panel right). G7 possesses polar and lateral flagella (Figure 1B), and could swim in 0.3% agar and swarm in 0.5% agar (Figure 1C).

### Phylogenetic Analysis of G7

**Figure 2:**
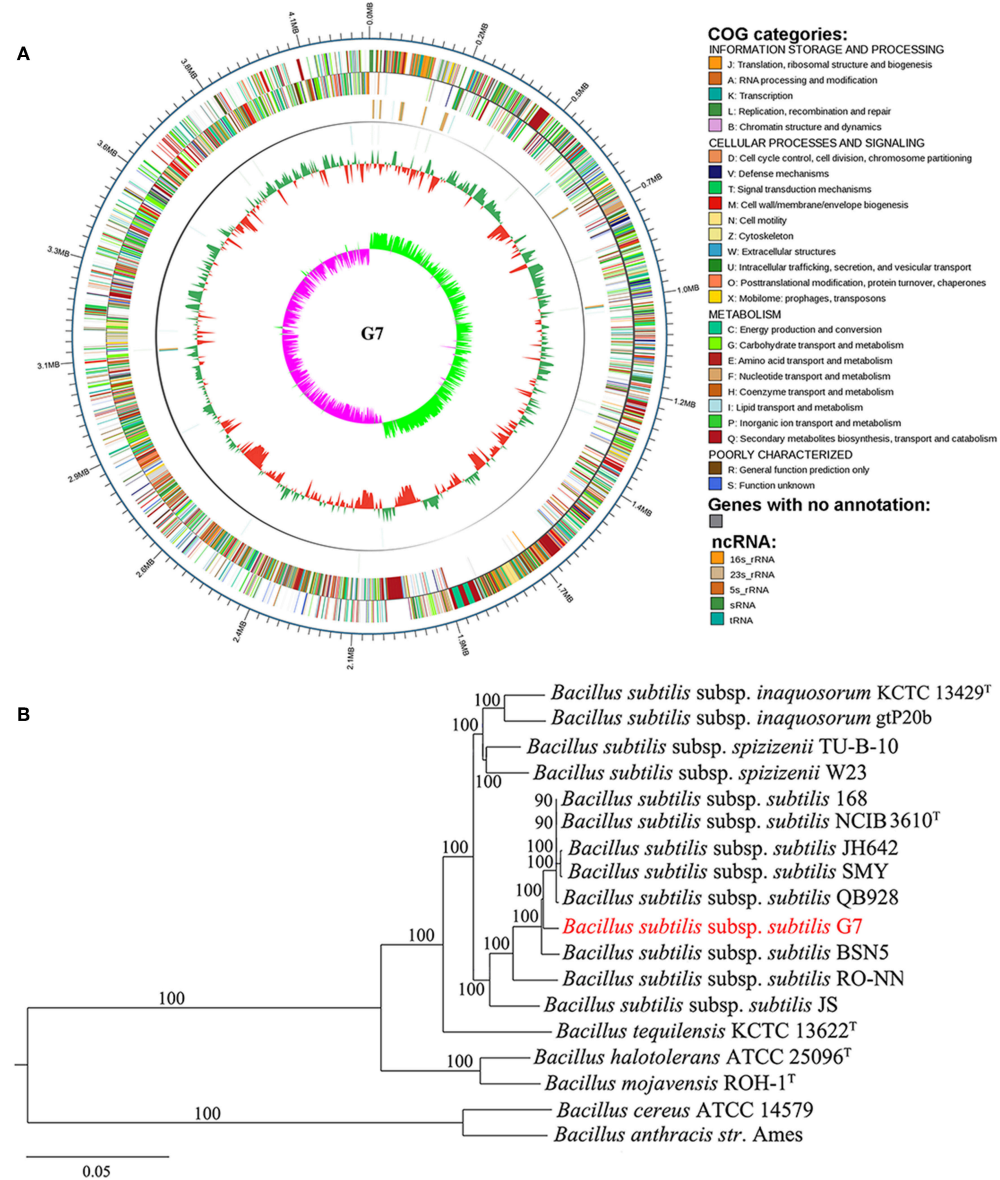
Circle genome map **(A)** and core genome phylogeny **(B)** of G7. **(A)** The scale on the outside of the circles indicates the size of G7 genome; the chromosome is represented by circles ranging from 1 (outer) to 4 (inner). Circle 1, the genes annotated by COG database (indicated by colors other than gray) and genes with no annotation (indicated by gray color); Circle 2, ncRNA, with colors indicating different types of ncRNA as shown in the figure; Circle 3, GC content; Circle 4, GC skew. **(B)** Neighbor-joining tree was constructed based on 1,113 highly conserved orthologous genes of 18 annotated genomes of *Bacillus*. Each node number represents the percentage of bootstrap support from 1,000 resampled datasets. The scale bar represents 0.05 substitutions per site.

To facilitate the study of G7, the genome of this strain was sequenced (Figure 2A). Comparative average nucleotide identity (ANI) analysis with available bacterial genomes in Integrated Microbial Genomes (IMG) database showed that the genome of G7 is most closely related to that of many sequenced strains of *B. subtilis* subsp. *subtilis*, and the ANI values between strain G7 and these strains are all higher than the hypothesized species demarcation threshold value of 95% (Table S2), which is generally accepted for species delineation (Richter and Rosselló-Móra, 2009). The 16S rRNA gene sequence similarity between strains G7 and *B. subtilis* subsp. *subtilis* QB928 is 99.80%, which is clearly above the threshold (97%) of 16S rRNA gene sequence similarity for species delineation (Richter and Rosselló-Móra, 2009). There are 1113 orthologous genes highly conserved in the members of 18 *Bacillus* species (Table S1) (Note: within these 18 *Bacillus* sp. strains, only a subset of sequenced *B. subtilis* subsp. *subtilis* strains were considered for analysis because the genome of different *B. subtilis* subsp. *subtilis* strains share high similarities; BSP1, 6051-HGW, BAB-1 and many other strains were not included in the analysis). In the genome tree based on the 1113 orthologous genes (Figure 2B), the *B. subtilis* species formed a group, which was separated from that formed by *Bacillus cereus* and *Bacillus anthracis*, and strain G7 was a member of *B. subtilis* subsp. *subtilis*.

### General Features of the G7 Genome

The general features of the G7 genome are summarized in Table 1 and Figure 2A. The genome is composed of one circular chromosome of 4,216,133 base pairs (bp) with an average GC content of 43.72%. The coding region accounts for 89.13% of the chromosome and was predicted to contain 4,416 genes which are distributed along both strands. Of the 4,416 genes, 1,215 (27.5%) could not be annotated; the remaining 3,201 (72.5%) genes could be annotated with known or predicted functions in 25 different COG categories, including those for transcription (category K, 10.3%), amino acid transport and metabolism (category E, 10.15%), carbohydrate transport and metabolism (category G, 9.74%), translation, ribosomal structure and biogenesis (category J, 7.18%), and cell wall/membrane/envelope biogenesis (category M, 7.06%). Ten sets of 23S, 5S, and 16S ribosomal RNA operons, 86 tRNA genes, and 14 sRNA (Table S3) were identified. The 10 rRNA operons exhibit very high similarity (>99.9%). Sixteen Genomic Islands (GIs) were found in the genome (Table S4), comprising 236,853 bp and 5.61% of the genome. The GIs vary very differently in size and gene content. G7 also contains 50 tandem repeat regions, 41 mini-satellites, one microsatellite, and 42 transposons.

**Table 1:** General features of *Bacillus subtilis* subsp. *subtilis* G7.

**Category**	**Characteristics**
Genome size (bp)	4,216,133
GC content (%)	43.72
Gene number	4,416
Coding region (bp)	3,757,905
Coding percentage (%)	89.13
Average gene length (bp)	851
Genes assigned to COG categories	3,201
tRNA genes	86
rRNA operons	10
Other regulatory ncRNAs	14
Genomic islands	16

### Comparative Genome Analysis Between G7 and its Close Homolog

**Figure 3:**
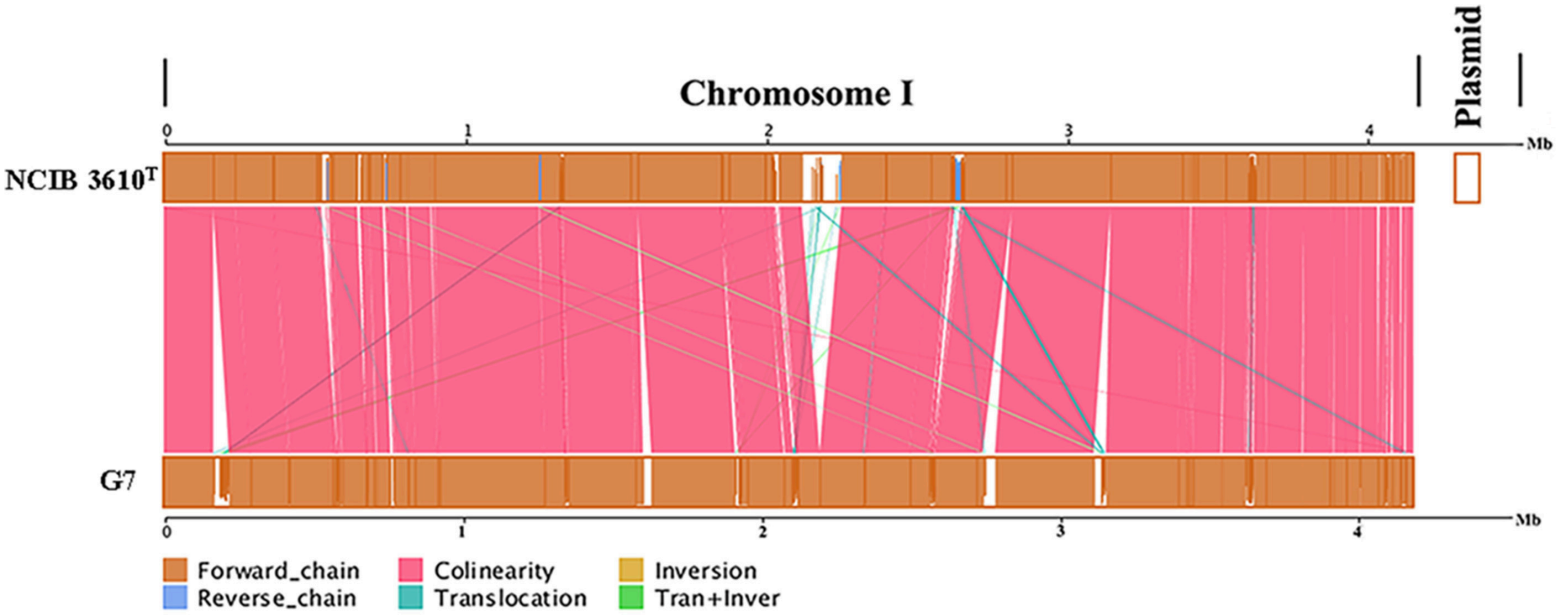
Genome alignments for G7 and NCIB 3610^T^ using MUMmer. Upper and lower axes of linear synteny graph are constructed after the same proportion of size reduction in length of both sequences. According to BLAST results, each pair nucleic acid sequence of the two alignments is marked in the coordinate diagram according to its position information, and the height of the filled color in the block indicates similarity of sequence alignment. The color of the lines between the two axes indicates the type of comparison.

**Figure 4:**
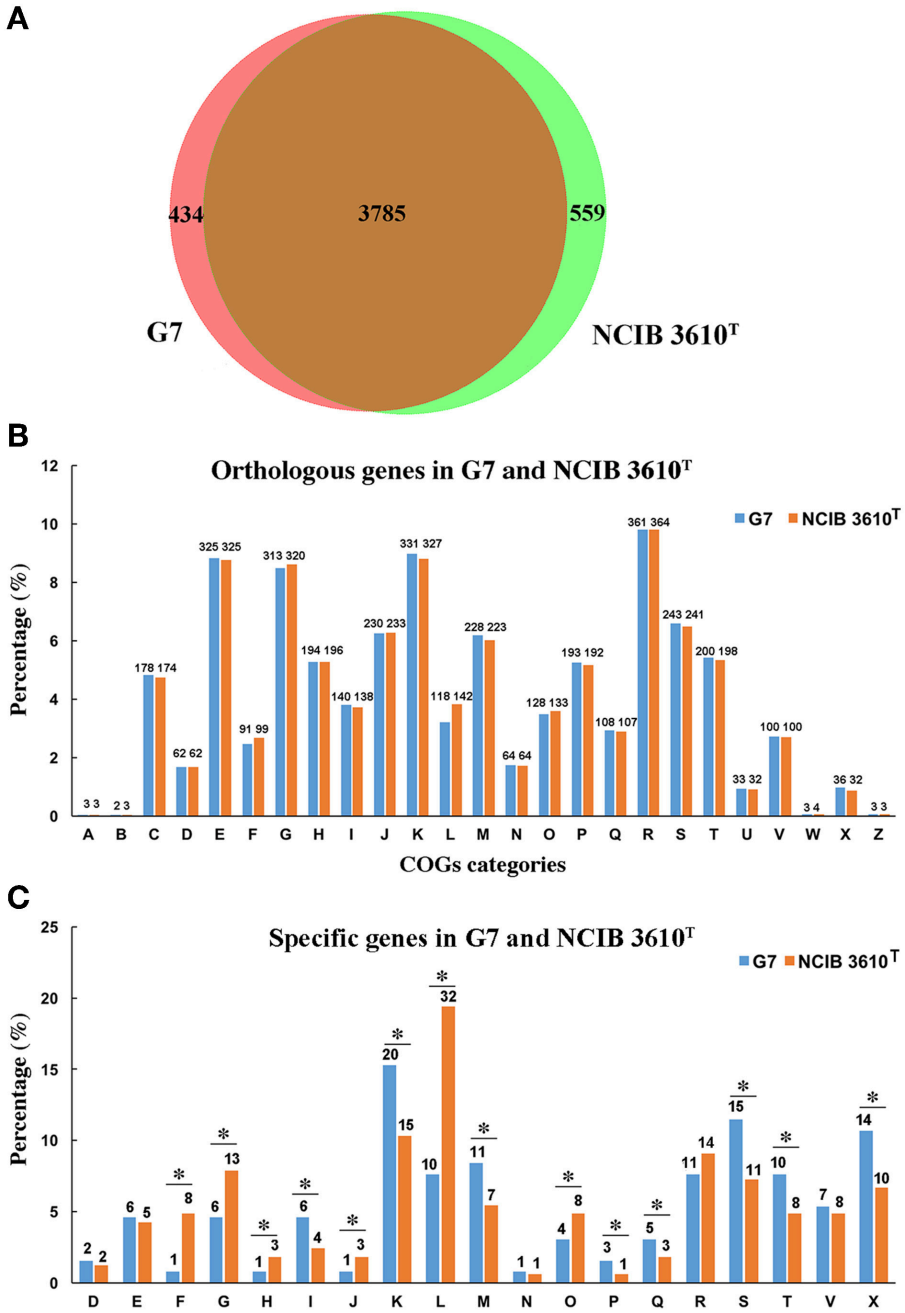
Comparison of the gene content of G7 and NCIB 3610^T^. **(A)** A Venn diagram of the orthologous and specific genes in G7 and NCIB 3610^T^. **(B)** Orthologous genes assigned to COG categories in each strain. **(C)** Specific genes assigned to COG categories in each strain. The alphabetic codes represent COG functional categories as shown in Figure 2. Asterisks indicate differences of more than 20%.

The genome of G7 was compared to that of its most closely related strain, *B. subtilis* subsp*. subtilis* NCIB 3610^T^, which was proposed to be the true wild type strain (Srivatsan et al., 2008). G7 genome is smaller and devoid of the plasmid present in NCIB 3610^T^ (Figure 3); the majority of genes in the two genomes exhibit strong collinearity and high sequence similarities; however, many genomic translocations, inversions, and insertions occur in G7 genome. G7 and NCIB 3610^T^ share 3785 orthologous genes, accounting for 89.7 and 87.13% of all the genes of G7 and NCIB 3610^T^, respectively, and contain 434 and 559 specific genes, respectively (Figure 4A). Among the 434 specific genes of G7, 146 (33.6%) and 288 (66.4%) were distributed in GIs and in “normal” coding regions, and no gene was found in repeated sequences. Most of the specific genes were annotated as hypothetical proteins and had no putative functions. Compared to NCIB 3610^T^, which has 8 GIs, G7 has twice the amount of GIs. GI_02, GI_03, GI_05, GI_10, and GI_13 carry genes encoding integrases, transposases, or phage portal proteins, GI_06 and GI_16 contain genes encoding ABC transporters; however, no GI genes were predicted to be involved in virulence. Many genes (47%) in the GIs of G7 are specific genes that do not have orthologs in NCIB 3610^T^, and more than 100 genes (42.5%) were predicted to encode hypothetical proteins (Table S4). COG analysis revealed that the distributions of the orthologous genes of the two strains in the COG functional classes were similar with respect to the total numbers of protein-coding genes (Figure 4B). However, with respect to the genes unique to each strain, G7 has higher proportions of specific genes belonging to the COG categories of K (transcription), S (function unknown), X (mobilome: prophages, transposons), M (cell wall/membrane/envelope biogenesis), I (lipid transport and metabolism), T (signal transduction mechanism), Q (secondary metabolite biosynthesis, transport, and catabolism), and P (inorganic ion transport and metabolism). In addition, G7 possesses lower proportions of specific genes belonging to the COG categories of F (nucleotide transport and metabolism), G (carbohydrate transport and metabolism), H (coenzyme transport and metabolism), J (translation, ribosomal structure, and biogenesis), L (dna replication, recombination, and repair), and O (posttranslational modification, protein turnover, and chaperones) (Figure 4C).

### Virulence-Associated Genes in G7 Genome

Many putative virulence genes were identified in G7 genome (Table 2), which were associated with toxins, adhesion, invasion, dissemination, anti-phagocytosis, and intracellular survival. All the predicted virulence genes in G7 are also present in the genome of NCIB 3610^T^. Among the toxins are *hlyIII* (GM002238) encoding hemolysin III, and *cylR2* encoding a protein similar to the virulence-contributing cytolysin of *Enterococcus faecali*s (Shankar et al., 2004). Among the adhesins are an adherence-related protein, endopeptidase ClpC, and a homolog of the fibronectin-binding protein FbpA of *Listeria monocytogenes*, which is the adhesin responsible for infectivity (Osanai et al., 2013). The genetic organization of the capsule genes *capA, capC, capB*, and *capD* (*GM003849-GM003851* and *GM002061*) in G7 is the same as that in *B. anthracis* str. Ames Ancestor (GenBank accession no. PRJNA10784), in which the *cap* operon is essential to virulence (Makino et al., 2002). G7 also possesses a hyaluronic acid capsule related gene (*GM003824*) similar to the *hasC* of *Streptococcus pyogenes*, which contributes to anti-phagocytosis, adherence, and tissue invasion (Ashbaugh et al., 1998; Bisno et al., 2003). Among the genes of the category of Intracellular Survival are *lplA1* (*GM001144*), which encodes a lipoate protein ligase necessary for efficient intracellular proliferation of *L. monocytogenes* (O'Riordan et al., 2003); *lspA* (*GM001724*), which encodes a lipoprotein signal peptidase required for the intracellular multiplication and survival of *Mycobacterium tuberculosis* in macrophages (Rampini et al., 2008; Pathak et al., 2015); *sodA* (*GM002170*) and *sodB* (*GM002589*), which were important for the intracellular survival and transmission of *M. tuberculosis* and *L. pneumophila* (Sadosky et al., 1994; Smith, 2003). A number of genes associated with iron acquisition were found in G7, including enterobactin, mycobactin, pyoverdine, pyochelin, and Fe-transport operon (FbpABC) (Table 2), which are known to promote the growth and invasion of some pathogens (Strange et al., 2011; Cassat and Skaar, 2013; Poppe et al., 2018; Qi and Han, 2018).

**Table 2:** Putative virulence factors of strain G7 and NCIB 3610^T^.

**Virulence genes**	**Annotation**	**Gene ID (G7)**	**Identity (%)**	**Gene ID (NCIB 3610^**T**^)**	**Identity (%)**
**TOXIN**					
*hlyIII*	Hemolysin III	GM002238	69.31	002367	68.1
*cylR2*	Cytolysin	GM001003	40.91	000972	40.9
**ADHERENCE**					
*htpB*	Hsp60	GM000708	84.44	000629	59.68
*fbpA*	FbpA	GM001743	51.66	001707	52.2
*clpC*	ClpC	GM000091	78.98	000092	79
**ESCAPE**					
*capA*	Capsule	GM003849	89.36	003872	88.21
*capC*	Capsule	GM003850	78.38	003873	79
*capB*	Capsule	GM003851	83.7	003874	83.2
*capD*	Capsule	GM002147	76	001999	78.1
*cap8B*	Capsule	GM003887	43.19	003789	42.8
*cap8J*	Capsule	GM004394	43.27	004407	42.3
*cap8D*	Capsule	GM003681	54.3	003589	53.5
*cpsD*	Capsule	GM003682	42.94	003590	42.77
*cpsE*	Capsule	GM003814	40.51	003718	40.29
*cps4l*	Capsule	GM003820	63.51	003723	62.34
*hasC*	Hyaluronic acid capsule	GM003824	55.75	003843	63.2
**INTRACELLULAR SURVIVAL**					
*lplA1*	LplA1	GM001144	65.26	001112	65.3
*lspA*	Lsp	GM001724	57.14	001688	57.1
*panD*	PanC/PanD	GM002304	53.78	002431	58.7
*panC*	PanC/PanD	GM002305	40.79	002432	43.7
*SodA*	SodA	GM002170	43.15	002105	45.7
*SodB*	SodB	GM002589	53.37	002710	53.4
**IRON UPTAKE**					
*fbpC*	FbpC	GM000152	40	002599	40.1
*fbpA*	FbpA	GM001743	51.66	001707	52.2
*fepC*	Enterobactin	GM000858	46.99	000785	46.75
*fbpABC*	FbpABC	GM002478	40.08	002510	40.06
*mbtH*	Mycobactin	GM003430	56.45	003338	56.17
*pvdD*	Pyoverdine	GM003431	43.21	003439	43.1
*entB*	Enterobactin	GM003432	48.83	003452	48.8
*pchD*	Pyochelin	GM003433	54.81	003453	60
*entA*	Enterobactin	GM003435	42.64	003343	42.32
**GENERAL INFECTION**					
*aur*	Aureolysin	GM001636	46.98	001202	41.6
*mprA*	MprAB	GM001477	46.22	001381	45.75
*mprB*	MprAB	GM004336	40.54	001381	40.12
*vscN*	TTSS	GM001802	52.61	001691	51.92
*hspR*	HspR	GM001927	40.3	001891	40.3
*sigA*	SigA	GM002608	62.38	002730	50.5
*relA*	RelA	GM002831	44.35	002987	44.5
*clpP*	ClpP	GM003700	77.6	003727	76.71
*ureA*	Urease	GM003930	61.16	003951	61.5
*ureB*	Urease	GM003931	48.28	003952	49.1
*ureC*	Urease	GM003932	57.14	003952	56.2
*narL*	Nitrate reductase	GM003997	40.99	004021	43.1
*narH*	Nitrate reductase	GM003999	56.73	004023	59.1
*narG*	Nitrate reductase	GM004000	49.79	004024	50.1

Many other virulence factors have also been identified, such as aureolysin, which is known to facilitate serum resistance and the spread of pathogens (Labreuche et al., 2010; Zhou et al., 2015), MprA, MprB, and MprP, which are required for establishment and maintenance of persistent infection in *M. tuberculosis* (Zahrt and Deretic, 2001; Zahrt et al., 2003).

### Analysis of the Virulence Potential of G7

#### *In vivo* Virulence Analysis

**Figure 5:**
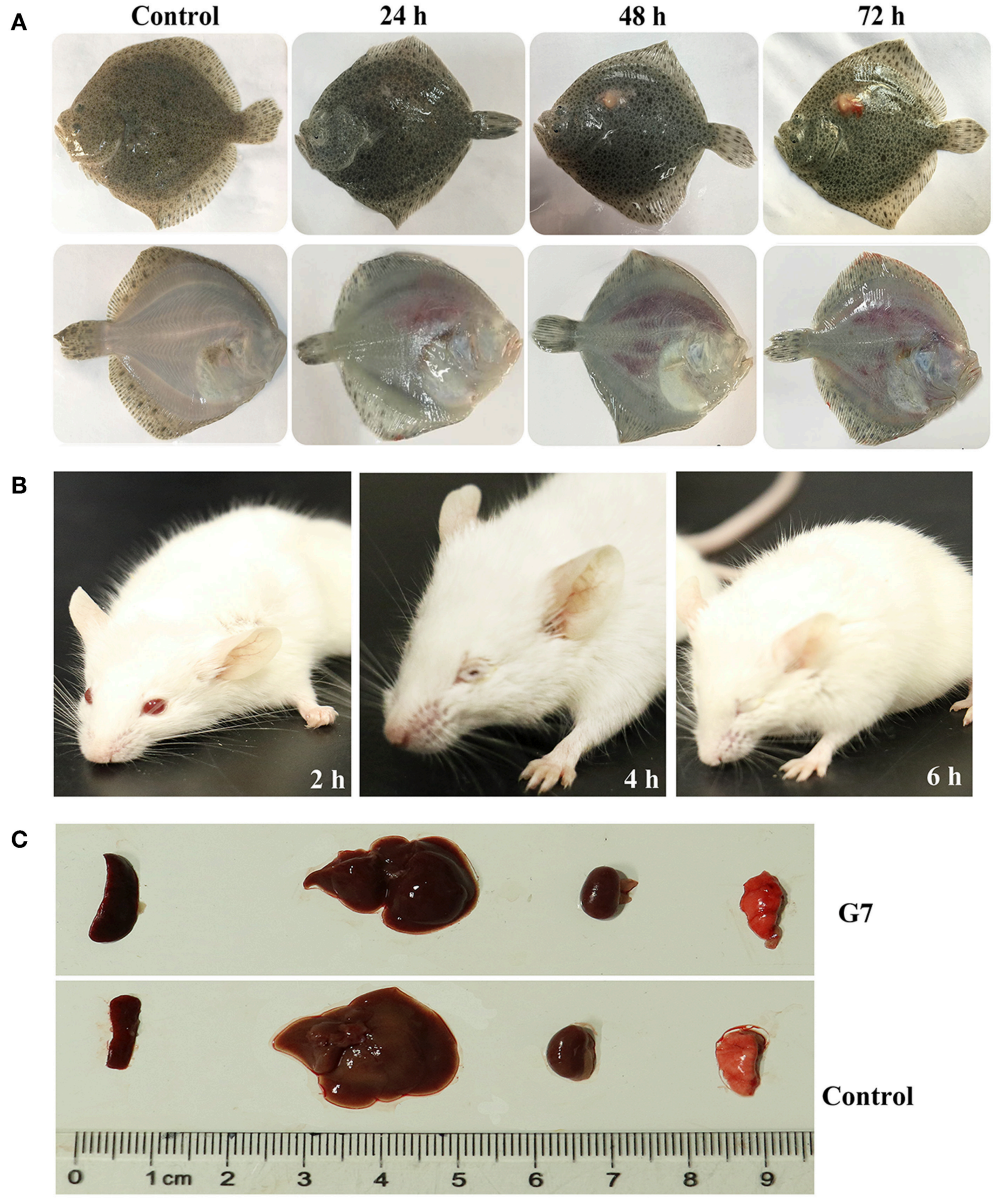
Symptoms of G7-infected animals. **(A)** Turbot were infected with or without (control) G7 and observed at different hours after infection. **(B)** G7-infected and uninfected (control) mice were observed at different hours post infection. **(C)** Organs of G7-infected and uninfected (control) mice were compared. The result is one representative of three mice.

**Figure 6:**
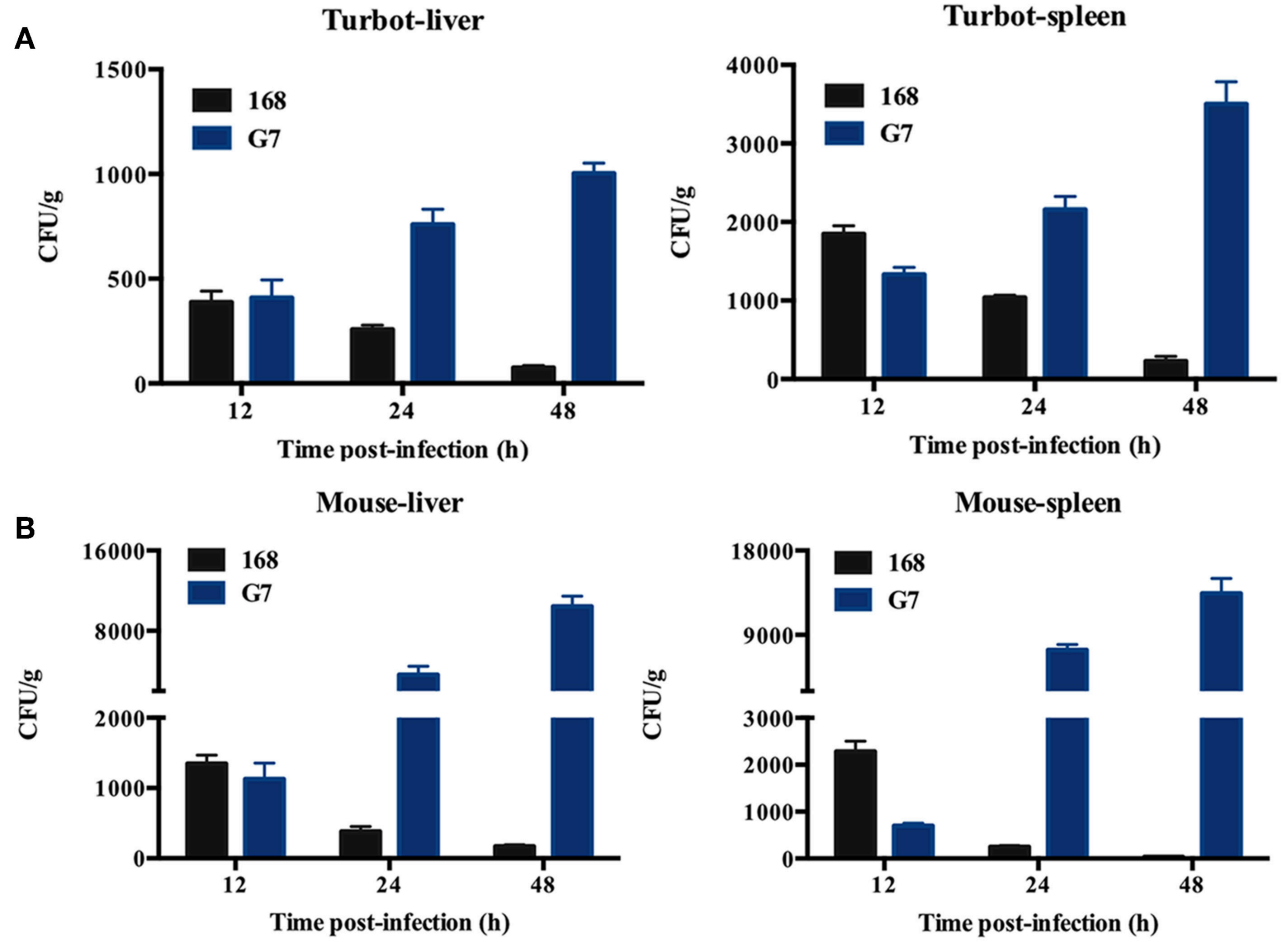
Dissemination of G7 in fish and mice tissues. Turbot **(A)** and mice **(B)** were inoculated with G7 or strain 168 (control), and bacterial recovery from the tissues was determined at different time points. The results are the means of three experiments and shown as means ± SEM.

The LD_50_ of G7 in lower (turbot and tongue sole) and higher (mice) vertebrates were determined by i.m. (for fish) and i.p. (for mice) infection. In comparison, the virulence potential of *Bacillus subtilis* subsp. *subtilis* NCIB 3610^T^ and *Bacillus subtilis* subsp. *subtilis* 168 were also determined. The results showed that the LD_50_ of G7 in turbot, tongue sole, and mice were 3.2 × 10^5^ CFU/g, 3.2 × 10^5^ CFU/g, and 5.4 × 10^5^ CFU/g, respectively; the LD_50_ of NCIB 3610^T^ in turbot and mice were 4.55 × 10^5^ CFU/g and 6.27 × 10^5^ CFU/g, respectively; the LD_50_ of strain 168 in turbot and mice were 2.93 × 10^6^ CFU/g and 8.45 × 10^6^ CFU/g, respectively. Turbot infected with G7 at the dose of 5 × 10^5^ CFU/g exhibited severe skin ulcer/lesion and hemorrhage (Figure 5A). When infecting mice at the dose of 2.5 × 10^7^ CFU/g, G7, NCIB 3610^T^, and strain 168 all caused 100% mortality within 24 h; G7-infected mice developed symptoms of shaking and arching of the back at 2 hpi, and secreting white substances from the eyes at 4 hpi; the mice began to die after 6 hpi (Figure 5B); moribund mice showed enlargement of spleen and congestion of liver (Figure 5C). When infecting mice at the dose of 2.5 × 10^6^ CFU/g, G7 and NCIB 3610^T^ caused 70% and 60% mortality, respectively, with death beginning to occur on the third day, whereas strain 168 induced no mortality et al (100% survival of the infected animal) (Figure S3). Tissue dissemination analysis showed that following muscle injection into turbot, G7 was detected in liver and spleen, with bacterial numbers increasing with time; strain 168 was also detected in fish tissues, however, the numbers of strain 168 were lower than G7 and decreased sharply with time (Figure 6A). Similar results were observed with bacterial recoveries from G7- and strain 168-infected mice (Figure 6B). No bacteria were detected in the blood of the infected mice or fish.

### Intracellular Replication in Host Phagocytes

**Figure 7:**
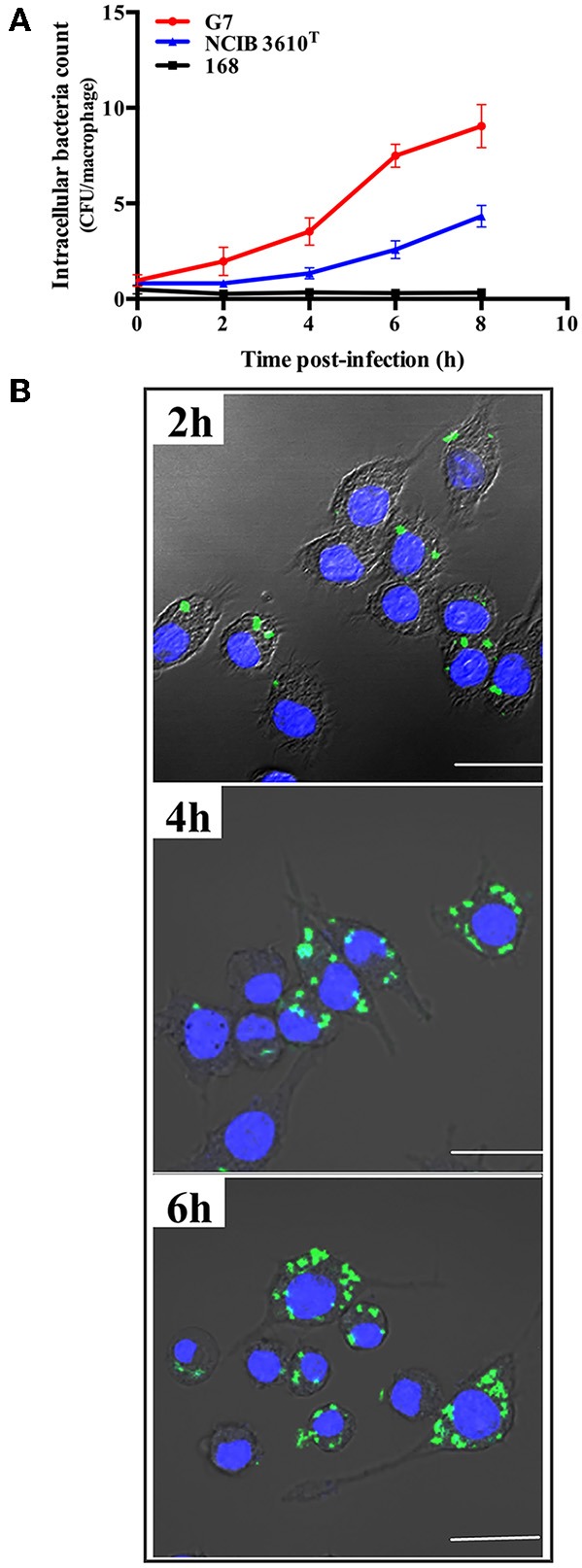
Replication of G7 in macrophages. **(A)** RAW264.7 cells were infected with G7, NCIB 3610^T^, and strain 168 for 2 h, and extracellular bacteria were killed by antibiotics. The cells were cultured further for 2, 4, 6, and 8 h, and intracellular bacterial number was determined by plate count. The results are the means of three experiments and shown as means ± SEM. **(B)** Microscopic observation of RAW264.7 infected with G7 as above. G7 and RAW264.7 were stained with CFDA-SE (green) and Hoechst 33258 (blue), respectively. Bar, 10 μm.

Intracellular replication study showed that following incubation with RAW264.7, G7, NCIB 3610^T^, and strain 168 were all detected inside the cells within 2 hpi. The intracellular numbers of G7 and NCIB 3610^T^ increased with time, however, the number of G7 increased much faster and reached much higher amount than that of NCIB 3610^T^, whereas no apparent intracellular replication of strain 168 was detected (Figure 7A). Consistently, confocal microscopy observed intracellular presence and replication of G7 in RAW264.7 (Figure 7B). Similar invasion and intracellular replication of G7 in turbot PBL were also observed (data not shown).

### Serum Resistance and Hemolytic Activity

**Figure 8:**
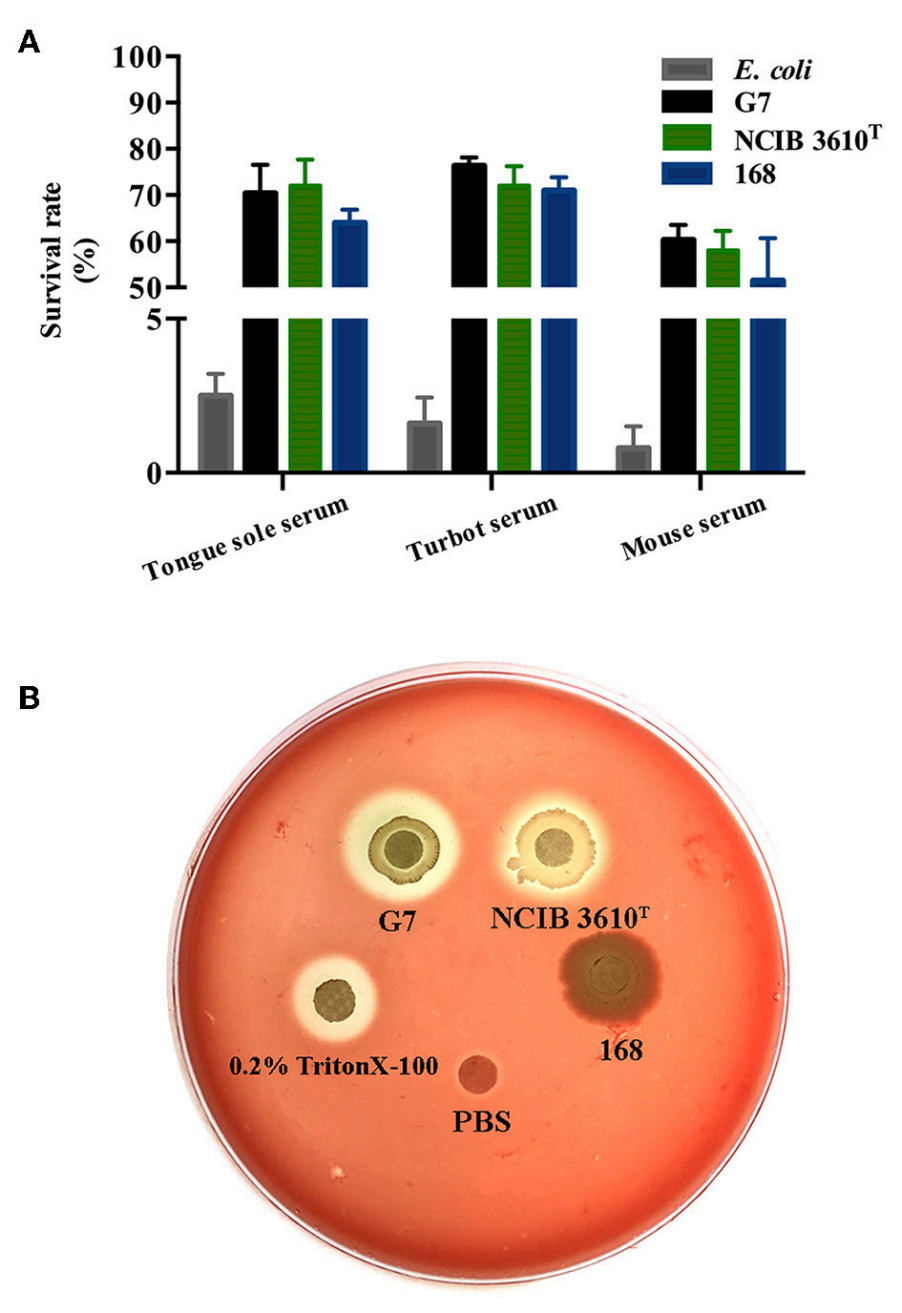
Serum resistance **(A)** and hemolytic activity **(B)** of G7. **(A)** G7, NCIB 3610^T^, strain 168, and *Escherichia coli* were incubated with or without (control) serum from tongue sole, turbot, or mouse, and bacterial survival was determined by plate count. The survival rate was expressed as (number of cells surviving serum treatment/number of cells in control treatment) × 100%. The data are the means of three experiments and shown as means ± SEM. **(B)** G7, NCIB 3610^T^, strain 168, 0.2% Triton X-100, and PBS were spotted onto the filter discs in rabbit blood agar plate, and the plate was observed for hemolytic halo after 24 h incubation.

Following incubation with the sera of tongue sole, turbot, and mice, the survival rates of G7 were 70.38, 76.3, and 60.28%, respectively; the survival rates of NCIB 3610^T^ were 72.3, 71.6, and 58%, respectively; the survival rates of strain 168 were 64.3, 71.9, and 51.5%, respectively; whereas the survival rates of *E. coli* DH5α, a non-virulent laboratory strain, after the same serum treatment were 2.5, 1.6, and 0.8%, respectively (Figure 8A). Hemolytic analysis indicated that G7 and NCIB 3610^T^, but not strain 168, were able to cause lysis of rabbit red blood cells (Figure 8B).

## Discussion

In this study, we examined the biological, genomic, and virulence characteristics of G7 from Iheya North hydrothermal field. Phylogenomic analysis indicated that G7 belongs to the *B. subtilis* subsp. *subtilis* species. It is of note that in the genome-based phylogenetic tree, the clade containing G7 was separated from that containing the pathogenic strains of *B. anthracis str*. Ames and *B. cereus* ATCC 14579, suggesting that G7 likely possesses some unique genetic characteristics that distinguish it from other pathogenic members of the *Bacillus* genus. Comparative genomic analysis between G7 and the *B. subtilis* subsp*. subtilis* wild type strain NCIB 3610^T^ (Srivatsan et al., 2008) revealed that G7 contains many translocations, inversions, and insertions. This observation, together with the fact that G7 is rich in mobile genetic elements such as integrases, transposases, and bacteriophage-related proteins, indicates that G7 genome may have undergone some genetic alterations via phage infection, horizontal gene transfer, and genetic reshuffling, which may explain why much more GIs were identified in the genome of G7 than in that of NCIB 3610^T^. This hypothesis is in line with the thought that in oceanic environments, phages are an important factor in the transfer of small gene cassettes between hosts (Lindell et al., 2004). Compared to NCIB 3610^T^, G7 possesses more specific genes belonging to the COG categories of K, T, S, X, and I. The K and T genes have been found to be associated with the capacity of niche adaptation and regulation of metabolism and transporters for nutrient acquisition in marine bacteria (Thomas et al., 2008; Lauro et al., 2009). The S category of G7 contains mainly hypothetical protein genes in the GIs, and the high proportion of specific genes belonging to X (mobilome: prophages, transposons) in G7 is consistent with the observation of many mobile genetic elements in the genome. In Antarctica bacteria, the abundance of genes in the I category (lipid transport and metabolism) of COG was considered to be a survival strategy to increase bacterial membrane fluidity at low temperatures (Médigue et al., 2005). Compared to NCIB 3610^T^, G7 also shows decrease in the genes involved in COG categories of F, G, H, L, and O, which is similar to previous observations in other deep-sea bacteria (Wang et al., 2008). The decrease of genes related to carbohydrate (G), nucleotide (F), and coenzyme (H) transport and metabolism is consistent with the oligotrophic conditions of deep-sea environments (Pedersen, 1993), where many bacteria appear to derive energy primarily from amino acid metabolism, rather than from sugar fermentation (Bartlett, 2002; Hou et al., 2004).

To date, *B. subtilis* has been extensively studied, primarily as a model for cell differentiation and exploitation in the biotechnology industry (Hoa et al., 2001; Asgher et al., 2007). *B. subtilis* has received little clinical attention as it has been associated only with opportunistic infections of immunocompromised patients (Ihde and Armstrong, 1973; Reller, 1973). In our study, we found that G7 could replicate in eukaryotic cells and cause acute symptom and mortality in teleost and mice following artificial inoculation. NCIB 3610^T^, the close homolog of G7, exhibited roughly similar capacities. It remains to be examined whether the clinical characteristics induced by G7 were the result of bacterial pathogenicity. Compared to NCIB 3610^T^, G7 showed slight but distinct differences in LD_50_, host lethality, and replication ability in phagocytic cells, however, the biological significance of these differences between the two strains remains to be investigated.

Previous reports showed that some virulent *Bacillus* specie possess certain capacities, notably secretion of virulent proteins such as hemolysin, protease, phospholipase, toxin, and cytotoxin (Dixon et al., 2000; Senesi and Ghelardi, 2010; Ramarao and Sanchis, 2013; Jeßberger et al., 2015), swimming and swarming motility (Senesi et al., 2010), and intracellular survival and escape (Dixon et al., 2000). In our study, genes associated with motility, toxicity, adhesion, invasion, immune escape, and intracellular survival are present in G7 genome. For motility, many genes related to flagellar assembly were found in G7. Previous studies showed that flagellum-mediated motility is important to allow bacteria to move toward favorable environments and for increasing pathogen-host interaction (La Ragione et al., 2000; Krukonis and DiRita, 2003; Dons et al., 2004; van Asten et al., 2004; Duan et al., 2013). In G7, in agreement with the presence of flagellar genes, the bacteria exhibited polar and lateral flagella and showed apparent swimming and swarming capacities. With respect to toxins, it has been reported that the hemolysin and enterococcal cytolysin of *Bacillus* species could act as tissue destructive/reactive proteins and damage the integrity of cellular plasma membrane (Baida and Kuzmin, 1996; Shankar et al., 2004; Senesi and Ghelardi, 2010). In G7, hemolysin III and cytolysin genes were identified, and G7 exhibited apparent hemolytic activity, suggesting that these genes may play a role in internal tissue damage that led to the observed hemorrhage of G7-infected animals in our study. Although the virulence-associated genes with known functions are highly similar in G7 and NCIB 3610^T^, G7 contains many more genomic alterations (translocations, inversions, and insertions) and twice the amount of GIs than NCIB 3610^T^, and, more importantly, most GIs genes are G7-specific and do not have orthologs in NCIB 3610T. These results suggest that in addition to the predicted virulence genes common to both G7 and NCIB 3610^T^, the large amount of unique GI genes specific to G7 may also play a role in the lethality of G7 to fish and mice.

The ability of a pathogen to attach to host cells is essential to establish infection (Zhang and Stephens, 1992). In our study, we found that G7 possesses putative adhesion factors including FbpA, ClpC, and Hsp60, all which are known to mediate attachment to host cells (Garduño et al., 1998; Osanai et al., 2013). Once inside the host, the pathogen has to combat with host defense in various forms such as phagocytosis- and complement-mediated killing. Reports have shown that *S. pyogenes* and *B. anthracis* were able to utilize their hyaluronic acid capsules to resist phagocytosis (Ashbaugh et al., 1998; Makino et al., 2002), and *B. anthracis* could also escape from complement killing by aid of its capsule (Lindberg, 1999). In the case of G7, it contains capsule genes and the genes of LplA1, LspA, PanD, PanC, SodA, and SodB, which are related to intracellular infection. In addition, G7 also possesses genes encoding enterobactin, mycobactin, pyoverdine, pyochelin, and Fe-transport operon, which are responsible for iron uptake (Strange et al., 2011; Cassat and Skaar, 2013; Poppe et al., 2018; Qi and Han, 2018). Being an essential nutrient, iron is required for successful bacterial survival in host cells. As a result, during infection, the bacteria have to exert various strategies to compete with the host for iron (Wilson et al., 2016). The presence of abundant iron acquisition genes as well as other genes involved in intracellular survival probably accounts at least in part for the capacity of the G7 to replicate in host phagocytes.

In conclusion, we investigated for the first time the virulence potential of a *B. subtilis* strain from deep-sea hydrothermal field. Our results showed that following artificial injection into lower and higher vertebrate animals, G7 was capable of tissue dissemination and inducing host mortality, but the underlying mechanism is not clear. The genome of G7 contains a large amount of genes encoding putative virulence factors as well as hypothetical proteins with unknown functions, however, whether these factors and proteins have actually contributed to the lethal effect observed with G7 remains to be investigated by future studies.

## Ethics Statement

Experiments involving live animals conducted in this study were approved by the Ethics Committee of Institute of Oceanology, Chinese Academy of Sciences. All methods were carried out in accordance with the relevant guidelines, including any relevant details.

## Author Contributions

JZ and Q-LS obtained the deep sea sample and performed bacterial isolation. LS and H-JG conceived and designed the experiments. H-JG and J-CL performed the experiments and analyzed the data. H-JG and LS wrote the paper.

### Conflict of Interest Statement

The authors declare that the research was conducted in the absence of any commercial or financial relationships that could be construed as a potential conflict of interest.
