# Supplementary material for: A First Study of the Virulence Potential of a Bacillus subtilis Isolate From Deep-Sea Hydrothermal Vent
Source: Front Cell Infect Microbiol. 2019 May 31;9:183. doi: 10.3389/fcimb.2019.00183 (PMC6554283; doi:10.3389/fcimb.2019.00183)

**A first study of the virulence potential of a *Bacillus subtilis* isolate from deep-sea hydrothermal vent**

Han-Jie Gu^a,b,c^, Jian Zhang^a,b^, Jing-Chang Luo^a,b,c^, Qing-Lei Sun^a,b^, Li Sun^a,b^*

*^a^CAS Key Laboratory of Experimental Marine Biology, Institute of Oceanology, Center for Ocean Mega-Science, Chinese Academy of Sciences, Qingdao, China*

*^b^Laboratory for Marine Biology and Biotechnology, Pilot National Laboratory for Marine Science and Technology, Qingdao, China*

***^c^****College of Earth and Planetary Sciences, University of Chinese Academy of Sciences, Beijing, China*

*To whom correspondence should be addressed

Mailing address:

Li Sun

Institute of Oceanology

Chinese Academy of Sciences

7 Nanhai Road

Qingdao 266071, China

Phone: 86-0532-82898829

Email: [lsun@qdio.ac.cn](mailto:lsun@qdio.ac.cn)

Running title: A virulent *Bacillus* from deep-sea

**Supplemental data**

**Table S1.** Accession numbers of 18 genome sequences of *Bacillus* sp*.* strains in NCBI GenBank database.

| **Species** | **Strain** | **Accession number** |
| --- | --- | --- |
| *Bacillus subtilis* subsp. *subtilis* | G7 | CP029609 |
| *Bacillus subtilis* subsp. *subtilis* | NCIB 3610^T^ | CP020102.1 |
| *Bacillus subtilis* subsp. *subtilis* | 168 | [AL009126.3](https://www.ncbi.nlm.nih.gov/nuccore/AL009126.3) |
| *Bacillus subtilis* subsp. *subtilis* | JH642 | [CM000489.1](https://www.ncbi.nlm.nih.gov/nuccore/CM000489.1) |
| *Bacillus subtilis* subsp. *subtilis* | SMY | [CM000490.1](https://www.ncbi.nlm.nih.gov/nuccore/CM000490.1) |
| *Bacillus subtilis* subsp. *subtilis* | BSn5 | [CP002468.1](https://www.ncbi.nlm.nih.gov/nuccore/CP002468.1) |
| *Bacillus subtilis* subsp. *subtilis* | RO-NN-1 | [CP002906.1](https://www.ncbi.nlm.nih.gov/nuccore/CP002906.1) |
| *Bacillus subtilis* subsp. *subtilis* | JS | NC_017743.1 |
| *Bacillus subtilis* subsp. *subtilis* | QB928 | [CP003783.1](https://www.ncbi.nlm.nih.gov/nuccore/CP003783.1) |
| *Bacillus subtilis* subsp. *spizizenii* | TU-B-10^T^ | [CP002905.1](https://www.ncbi.nlm.nih.gov/nuccore/CP002905.1) |
| *Bacillus subtilis* subsp. *spizizenii* | W23 | [CP002183.1](https://www.ncbi.nlm.nih.gov/nuccore/CP002183.1) |
| *Bacillus subtilis* subsp. *inaquosorum* | KCTC 13429^T^ | AMXN01000001.1 |
| *Bacillus subtilis* subsp. *inaquosorum* | gtP20b | AEHM00000000 |
| *Bacillus* *tequilensis* | KCTC 13622^T^ | AYTO01000001.1 |
| *Bacillus mojavensis* | ROH-1^T^ | AFSI01000001.1 |
| *Bacillus halotolerans* | ATCC 25096^T^ | LPVF01000001.1 |
| *Bacillus anthracis* | str. Ames | NC_003997.3 |
| *Bacillus cereus* | ATCC 14579 | NC_004722.1 |

**Table S2**. ANI values of 9 *Bacillus subtilis* subsp. strains.

| **OrthoANI (%)** | **G7** | **QB928** | **NCIB 3610^T^** | **168** | **JH642** | **SMY** | **BSn5** | **RO-NN-1** | **JS** |
| --- | --- | --- | --- | --- | --- | --- | --- | --- | --- |
| **G7** | 100 | 98.77 | 98.73 | 98.71 | 98.69 | 98.69 | 98.35 | 98.02 | 95.40 |
| **QB928** |  | 100 | 99.96 | 99.96 | 99.92 | 99.92 | 98.44 | 98.10 | 95.40 |
| **NCIB 3610^T^** |  |  | 100 | 88.99 | 99.96 | 99.96 | 98.41 | 98.09 | 95.42 |
| **168** |  |  |  | 100 | 99.96 | 98.42 | 98.42 | 98.09 | 95.42 |
| **JH642** |  |  |  |  | 100 | 99.98 | 98.38 | 98.04 | 95.39 |
| **SMY** |  |  |  |  |  | 100 | 98.42 | 98.01 | 95.32 |
| **BSn5** |  |  |  |  |  |  | 100 | 97.91 | 95.28 |
| **RO-NN-1** |  |  |  |  |  |  |  | 100 | 95.41 |
| **JS** |  |  |  |  |  |  |  |  | 100 |

**Table S3**. Characteristics of the predicted sRNA of G7.

| **Site** | **Type** | **Start** | **End** | **Score** | **Strand** | **Annotation** |
| --- | --- | --- | --- | --- | --- | --- |
| Chr1 | sRNA | 9406 | 9340 | 66.7 | - | Bacillaceae-1 RNA; Threshold=40.00 |
| Chr1 | sRNA | 29932 | 29866 | 66.7 | - | Bacillaceae-1 RNA; Threshold=40.00 |
| Chr1 | sRNA | 160531 | 160465 | 61.1 | - | Bacillaceae-1 RNA; Threshold=40.00 |
| Chr1 | sRNA | 197704 | 197462 | 134.8 | - | BsrG; Threshold=40.00 |
| Chr1 | sRNA | 371606 | 371672 | 52.2 | + | Bacillaceae-1 RNA; Threshold=40.00 |
| Chr1 | sRNA | 528658 | 528572 | 78.8 | - | BsrC; Threshold=40.00 |
| Chr1 | sRNA | 589521 | 589607 | 91.3 | + | BsrC; Threshold=40.00 |
| Chr1 | sRNA | 823561 | 823627 | 49.6 | + | Bacillaceae-1 RNA; Threshold=40.00 |
| Chr1 | sRNA | 963900 | 963834 | 66.7 | - | Bacillaceae-1 RNA; Threshold=40.00 |
| Chr1 | sRNA | 1250630 | 1250744 | 120.5 | + | RNA Staph. aureus E; Threshold=40.00 |
| Chr1 | sRNA | 2139295 | 2139403 | 101.2 | + | BsrF; Threshold=40.00 |
| Chr1 | sRNA | 3121500 | 3121566 | 66.7 | + | Bacillaceae-1 RNA; Threshold=40.00 |
| Chr1 | sRNA | 3766930 | 3766864 | 60.9 | - | Bacillaceae-1 RNA; Threshold=40.00 |
| Chr1 | sRNA | 3822777 | 3822711 | 42.7 | - | Bacillaceae-1 RNA; Threshold=40.00 |

**Table S4**. Characteristics of the genomic islands found in the genome of G7.

| **GIs_id** | **Start** | **End** | **Size (bp)** | **No. of genes** | **No. of**  **specific gene** | **No. of**  **hypothetical protein** |
| --- | --- | --- | --- | --- | --- | --- |
| GI_01 | 114,842 | 151,705 | 36,864 | 50 | 0 | 1 |
| GI_02 | 159,348 | 173,903 | 14,556 | 16 | 13 | 8 |
| GI_03 | 177,151 | 197,299 | 20,149 | 36 | 22 | 17 |
| GI_04 | 214,188 | 230,063 | 15,876 | 11 | 8 | 7 |
| GI_05 | 581,992 | 600,962 | 18,971 | 23 | 12 | 7 |
| GI_06 | 609,095 | 619,646 | 10,552 | 14 | 1 | 4 |
| GI_07 | 1,141,640 | 1,149,274 | 7,635 | 7 | 0 | 0 |
| GI_08 | 1,709,431 | 1,718,025 | 8,595 | 8 | 0 | 1 |
| GI_09 | 1,740,984 | 1,746,841 | 5,858 | 7 | 0 | 0 |
| GI_10 | 1,928,097 | 1,953,452 | 25,356 | 37 | 20 | 25 |
| GI_11 | 2,117,166 | 2,137,648 | 20,483 | 29 | 20 | 24 |
| GI_12 | 2,580,572 | 2,598,110 | 17,539 | 25 | 18 | 17 |
| GI_13 | 2,762,754 | 2,773,619 | 10,866 | 17 | 12 | 6 |
| GI_14 | 2,801,536 | 2,807,922 | 6,387 | 11 | 8 | 3 |
| GI_15 | 4,033,307 | 4,038,833 | 5,527 | 9 | 9 | 9 |
| GI_16 | 4,119,509 | 4,131,147 | 11,639 | 10 | 3 | 3 |
|  | |  | | 310 | 146 (47%) | 132 (42.5%) |

**Figures S1**. Growth conditions of G7 at different NaCl concentrations. G7 was cultured in marine 2216E medium containing different concentrations of NaCl (1%-9%) for 3 days, and growth was observed.


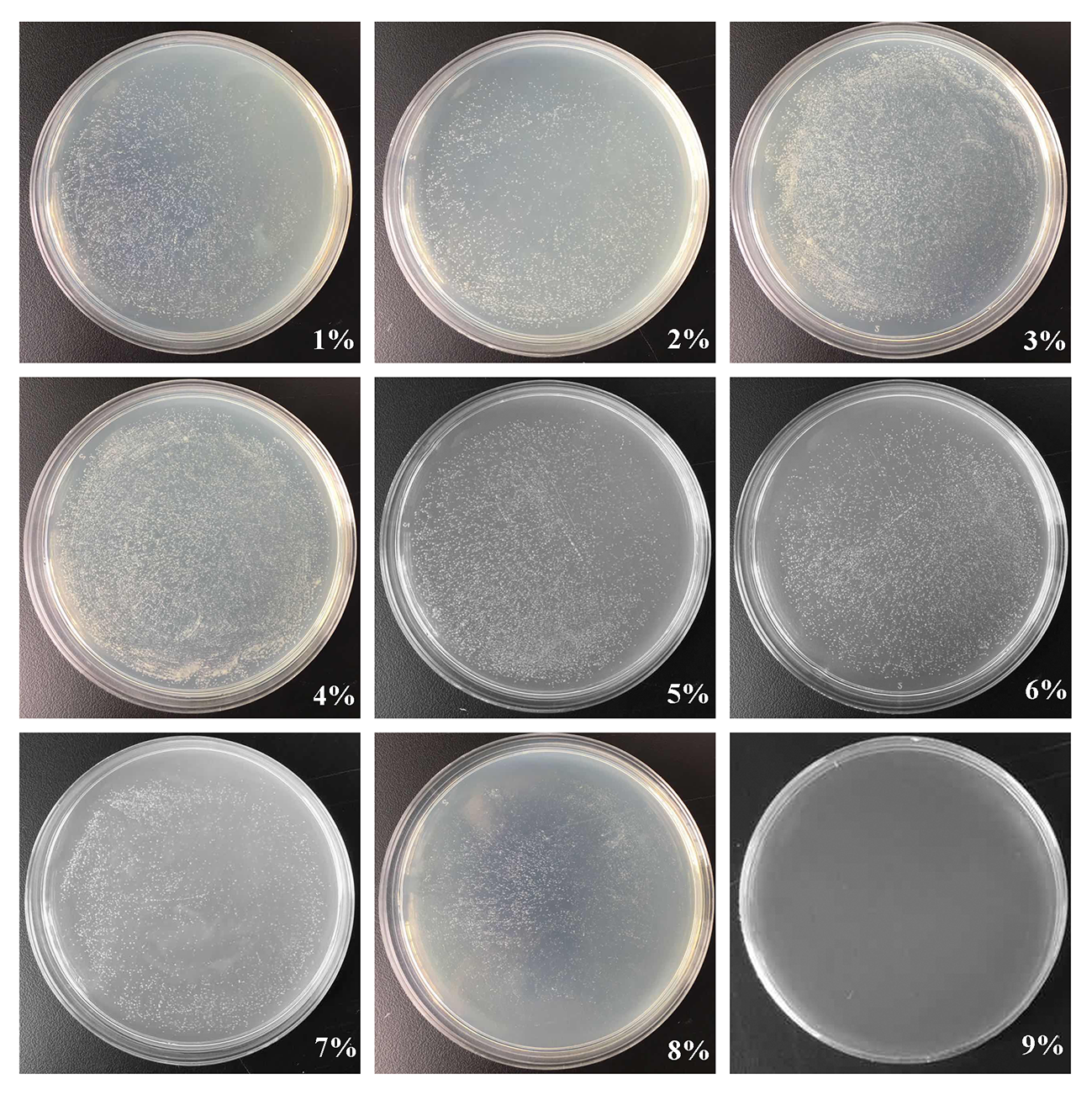


**Figures S2**. Growth analysis of G7 and NCIB 3610^T^ at different temperatures. G7 and NCIB 3610^T^ were cultured in marine 2216E and LB medium, respectively, and were measured for cell density at various time points. The results are the means of triplicate experiment and shown as means ± SEM.


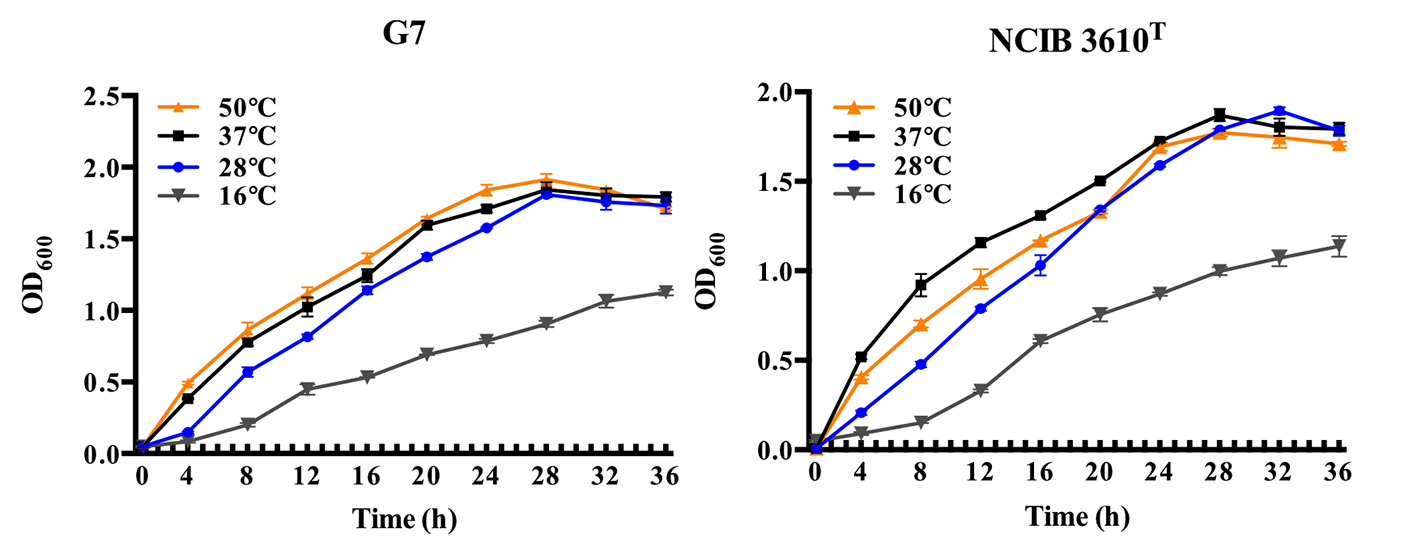


**Figure S3.** Survival of infected mice. Mice were infected with G7, NCIB 3610^T^, and strain 168 at the dose of 2.5 × 10^6^ CFU/g or treated with PBS (control) and monitored daily for survival. Significance between the survivals of the infected animals and the control animals was determined. ***P* < 0.01.


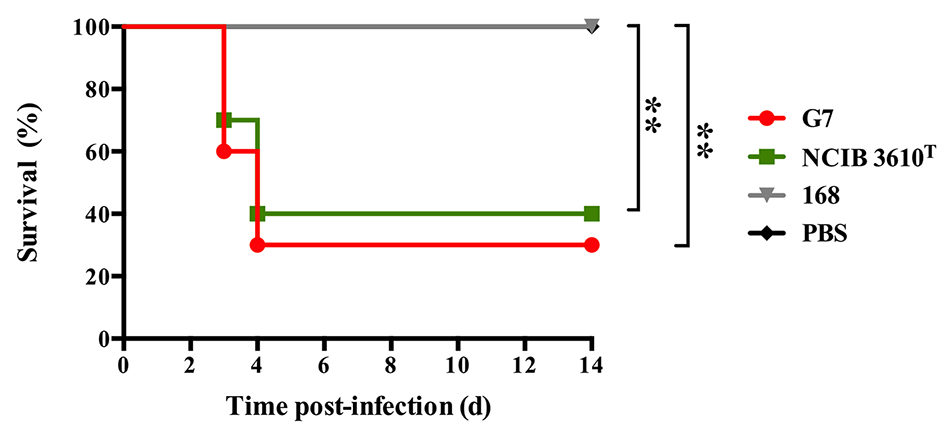

Supplement: Supplementary file 1 [file Data_Sheet_1.doc]
